# Human Plasma Butyrylcholinesterase Hydrolyzes Atropine: Kinetic and Molecular Modeling Studies

**DOI:** 10.3390/molecules29092140

**Published:** 2024-05-04

**Authors:** Aliya Mukhametgalieva, Showkat Ahmad Mir, Zukhra Shaihutdinova, Patrick Masson

**Affiliations:** 1 Laboratory of Biochemical Neuropharmacology, Kazan Federal University, Kremlevskaya Str. 18, 420008 Kazan, Russia; aliya_rafikovna@mail.ru (A.M.); shajhutdinova.z@mail.ru (Z.S.); 2 School of Life Sciences, Sambalpur University, Jyotivihar, Burla 768019, India; showkat@suniv.ac.in

**Keywords:** atropine, atropinesterase, butyrylcholinesterase, steady-state hydrolysis, molecular dynamics

## Abstract

The participation of butyrylcholinesterase (BChE) in the degradation of atropine has been recurrently addressed for more than 70 years. However, no conclusive answer has been provided for the human enzyme so far. In the present work, a steady-state kinetic analysis performed by spectrophotometry showed that highly purified human plasma BChE tetramer slowly hydrolyzes atropine at pH 7.0 and 25 °C. The affinity of atropine for the enzyme is weak, and the observed kinetic rates versus the atropine concentration was of the first order: the maximum atropine concentration in essays was much less than *K_m_*. Thus, the bimolecular rate constant was found to be *k_cat_*/*K_m_* = 7.7 × 10^4^ M^−1^ min^−1^. Rough estimates of catalytic parameters provided slow *k_cat_* < 40 min^−1^ and high *K_m_* = 0.3–3.3 mM. Then, using a specific organophosphoryl agent, echothiophate, the time-dependent irreversible inhibition profiles of BChE for hydrolysis of atropine and the standard substrate butyrylthiocholine (BTC) were investigated. This established that both substrates are hydrolyzed at the same site, i.e., S198, as for all substrates of this enzyme. Lastly, molecular docking provided evidence that both atropine isomers bind to the active center of BChE. However, free energy perturbations yielded by the Bennett Acceptance Ratio method suggest that the L-atropine isomer is the most reactive enantiomer. In conclusion, the results provided evidence that plasma BChE slowly hydrolyzes atropine but should have no significant role in its metabolism under current conditions of medical use and even under administration of the highest possible doses of this antimuscarinic drug.

## 1. Introduction

**Figure 1:**
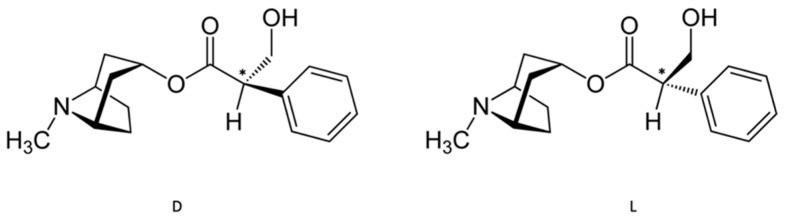
Atropine; and D and L isomers. Ester of tropic acid (3-hydroxy-2-phenylpropanoic acid) and α-tropan-3ol. C* are the chiral center.

Atropine, a major anticholinergic drug that is an agonist of muscarinic receptors, is a tropane ester like hyoscine and cocaine [1]. Atropine is a chiral molecule (Figure 1). The *S*-(-)-form has the most potent antimuscarinic properties, but only the racemic form of sulfate salt (CAS 5908-99-6) is used in medicine.

The problem of the fate of atropine in humans and, in particular, its hydrolysis in blood has been recurrently addressed for more than 80 years [2,3]. In human plasma, the enzyme-catalyzed hydrolysis of drug esters is mostly performed by carboxylesterases (CES) [4,5], butyrylcholinesterase (BChE, EC.3.1.1.8) [6], and albumin to some extent [7]. Although human plasma rapidly hydrolyzes (+)-cocaine (*k*_cat_ = 7500 min^−1^) and, much more slowly, (-)-cocaine (*k*_cat_ = 3.9 min^−1^) [8], there is no evidence that plasma BChE is involved in the hydrolysis of the bulkier tropane esters, atropine, and hyoscine (scopolamine) [1]. Yet, human plasma displays a small “atropine esterase” (EC.3.1.1.10) activity [9], and OP pesticides inhibit the metabolism of atropine in humans [10]. It is not known whether this atropine esterase activity is due to a single enzyme or to several serine esterases, including a carboxylesterase as in rabbit plasma [11]. However, atropine and hyoscine are clearly reversible ligands of BChE, causing either its allosteric activation or inhibition, depending on the reporter substrate and atropine concentration [12,13]. Thus, these tropane esters likely first bind to the peripheral anionic site (PAS) of the enzyme [14] and then may slide down the active site gorge of the enzyme for hydrolysis at the catalytic active center (CAC) [6]. It was, therefore, important to revisit the puzzling question about the possible participation of BChE in the catabolism of atropine. For this purpose, kinetic studies were performed with racemic atropine, using highly purified human plasma BChE. The highest concentration of atropine used for in vitro assays was much lower than the concentrations of atropine in plasma after the current therapeutic administration of atropine in humans. The results show unambiguously that both isomers of atropine bind to the BChE active center. BChE displays a small catalytic activity against atropine that may have no significant incidence on the catabolism of this drug. Indeed, the *K_m_* of BChE for atropine is much higher than the highest possible therapeutic doses of atropine. Although the catalytic behavior of BChE with this bulky substrate is complex, the results indicate that atropine is hydrolyzed at CAC residue S198 as other substrates of this enzyme.

## 2. Results

### 2.1. Steady-State Hydrolysis of Racemic Atropine Sulfate

**Figure 2:**
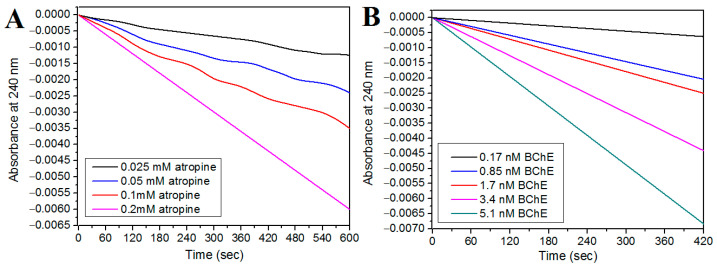
Steady-state hydrolysis of atropine by human BChE in 0.1 M phosphate buffer, pH 7.0 at 25 °C. (**A**) Hydrolysis as a function of atropine concentration [A] (grey line, 0.025 mM; red line, 0.05 mM; blue line, 0.1 mM; pink line, 0.2 mM) using [E] = 1.7 nM. (**B**) BChE-catalyzed hydrolysis rate of [A] = 0.1 mM at increasing enzyme concentration.

**Figure 3:**
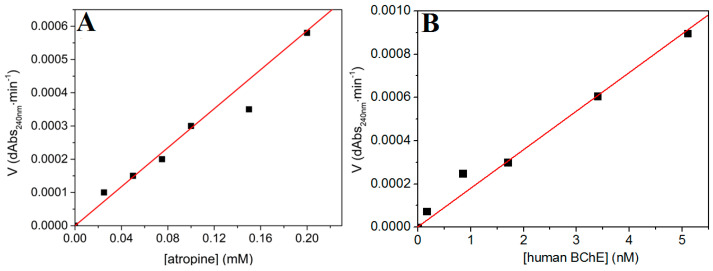
(**A**) Hydrolysis rate (−dAbs/dt) of racemic atropine (mM) by human BChE ([E] = 0.17 × 10^−8^ M; 1.7 nM) as a function of atropine concentration in 0.1 M phosphate pH 7.0 at 25 °C; r^2^ = 0.99, slope = 0.00293 ± 0.0001. (**B**) Hydrolysis rate (−dAbs/dt) of racemic atropine ([atropine] = 0.1 mM) by different concentrations ([E]) of human BChE ranging from 0.17 nM to 5.1 nM in 0.1 M phosphate pH 7.0 at 25 °C; r^2^ = 0.99; slope = 0.000179 ± 0.000007.

Kinetic studies showed that BChE slowly hydrolyzes atropine. Kinetics were performed at increasing concentrations in atropine, using a single enzyme concentration (Figure 2A and Figure 3A), and increasing enzyme concentration, using a single atropine concentration (Figure 2B and Figure 3B). The progress curves of BChE-catalyzed atropine hydrolysis (0.025; 0.050; 0.1; 0.2 mM) showed no burst or lag before the establishment of the steady state (Figure 2). This indicates no hysteretic catalytic behavior with this bulky substrate. Despite the low rate of hydrolysis, the linear dependence of the rate on the enzyme concentration clearly demonstrated that atropine is hydrolyzed by BChE (Figure 3B). It should be noted that atropine is stable in phosphate buffer and that no decay in absorbance at 240 nm was observed even at the highest atropine concentration used (0.2 mM). Also, because BChE-catalyzed hydrolysis of atropine is very slow, progress curves at the lowest atropine concentrations (0.025, 0.5, and 0.1 mM) are noisy.

However, a steady-state analysis of the hydrolysis kinetics showed a linear rate of hydrolysis versus atropine concentration [A] at least up to 0.2 mM, the highest atropine concentration we used (Figure 2A). Thus, atropine was hydrolyzed under first-order conditions (Equation (1)).

(1) $$v=\frac{k_{cat}}{K_{m}}\left\lfloor E\right\rfloor \left\lfloor A\right\rfloor$$

This result indicates that *K_m_* is much higher than 0.2 mM. The bimolecular rate constant was calculated from the slope of the plot (Figure 3A), *k_cat_*/*K_m_* = 7.7 ± 0.3 × 10^4^ M^−1^min^−1^.

**Scheme 1:**
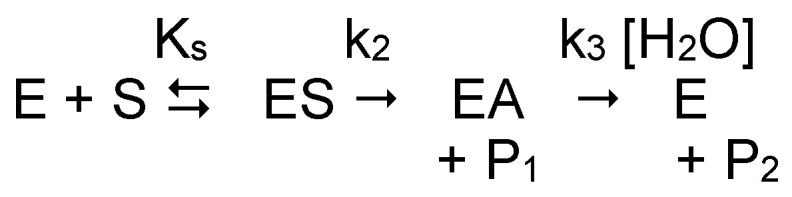
BChE-catalyzed hydrolysis scheme for low concentrations of esters.

Taking into account that the hydrolysis of atropine by BChE obeys the general mechanistic scheme for hydrolysis, at low concentrations, of ester substrates (S) by ChEs, i.e., the simple Michaelis–Menten mechanism, with a binding step (formation of complex ES) followed by two kinetic steps, acylation (*k*_2_) and deacylation (*k*_3_) (Scheme 1):

The bimolecular rate constant, *k_cat_*/*K_m_*, is equal to *k*_2_/*K_s_*. In the case of good ester substrates, it is established that, for the hydrolysis of ester substrates by ChEs, acylation and deacylation are partly rate-limiting (*k*_2_/*k*_3_ not very different) [15]. However, in the case of bulky substrates like cocaine, acylation is rate-limiting (*k*_2_ << *k*_3_). Such a situation must prevail for the hydrolysis of atropine, which is slightly bulkier than cocaine. Thus, a rough estimation of *k_cat_* and *K_m_* was attempted.

Because medicinal atropine is a racemic, the poor bimolecular rate constant for the degradation of atropine is much lower than *k_cat_*/*K_m_* for BChE-catalyzed hydrolysis of the most active isomer of cocaine, (+)-cocaine, for which *k_cat_*/*K_m_* = 9.0 × 10^7^ M^−1^ min^−1^ (while for (-)-cocaine, *k_cat_*/*K_m_* = 4.4 × 10^4^ M^−1^ min^−1^) [8]. Although atropine and cocaine are tropane alkaloids with related acyl moieties, atropine is bulkier than cocaine, and their stereochemistry shows differences [16]. Thus, taking into account the stereochemistry of atropine and cocaine, it can be assumed that, as for cocaine, one atropine stereoisomer is preferentially hydrolyzed by BChE. The following in silico work in Section 2.3 allowed us to decide which isomer is preferentially hydrolyzed. However, we should point out that the chiral separation of L and D atropine is possible [17], and that working on pure atropine enantiomers, it would be possible to directly monitor the preferential stereoselectivity of BChE for the hydrolysis of this tropane ester.

Starting from this point, a rough estimate of catalytic parameters can reasonably be attempted.

Old studies showed that atropine acts as a competitive inhibitor of BChE-catalyzed hydrolysis of acetyl- and butyryl-choline esters with a *K_i_* of the order of 0.15–0.5 mM [13]. Thus, *K_i_* may be regarded as being of the order of *K*_s_, the dissociation constant of atropine BChE complex in Scheme 1. Then, because (Equation (2)), eIt follows that k_2_ is of the order of 11.5–38.5 min^−1^.

(2) $$\frac{k_{cat}}{K_{m}}=\frac{k_{2}}{K_{s}}$$

Then, as for the BChE-catalyzed hydrolysis of cocaine, where acylation is the rate-limiting step (*k*_2_ << *k*_3_), the acylation of BChE by atropine is rate-limiting. Thus, this value is close to the *k_cat_* value. Then, because the *P_2_* products of deacylation (benzoic acid for benzoylcholine and cocaine, and 3-hydroxy-2-phenylpropanoic acid (tropic acid) for atropine) are closely related, the deacylation rate constants for benzoyl choline, cocaine, and atropine are of the same order. *k*_3_ was determined as 19.800 min^−1^ from the kinetic study of BChE-catalyzed hydrolysis of benzoylcholine [18]. Then, taking this value as the highest possible value for deacylation of BChE reacting with atropine, the *k_cat_* for the hydrolysis of atropine may be estimated from Equation (3):

(3) $$k_{cat}=\frac{k_{2}}{1+\frac{k_{2}}{k_{3}}}≅k_{2}$$

Thus, *k_cat_* ranges between 11.5 and 38.5 min^−1^, and the *K_m_* estimated from Equation (2) ranges between 0.3 and 3.35 mM.

### 2.2. Irreversible Inhibition of BChE-Catalyzed Hydrolysis of Atropine and Butyrylthiocholine by Echothiophate

The irreversible inhibitory action of echothiophate (5 × 10^−8^ M) on the activity of BChE ([E] = 1.7 × 10^−9^ M) was performed under first-order conditions ([*E*] << [echothiophate]) to determine whether the inhibitory behavior of the enzyme is the same with both substrates. The concentrations of both reporter substrates, BTC and atropine, were low. The atropine concentration (0.2 mM) was below the estimated *K_m_* for this substrate, and the BTC concentration (0.05 mM) was slightly above *K_m_* for BTC [19]. Thus, under these conditions, the behavior of the enzyme residual activity was Michaelian with both substrates. 

**Figure 4:**
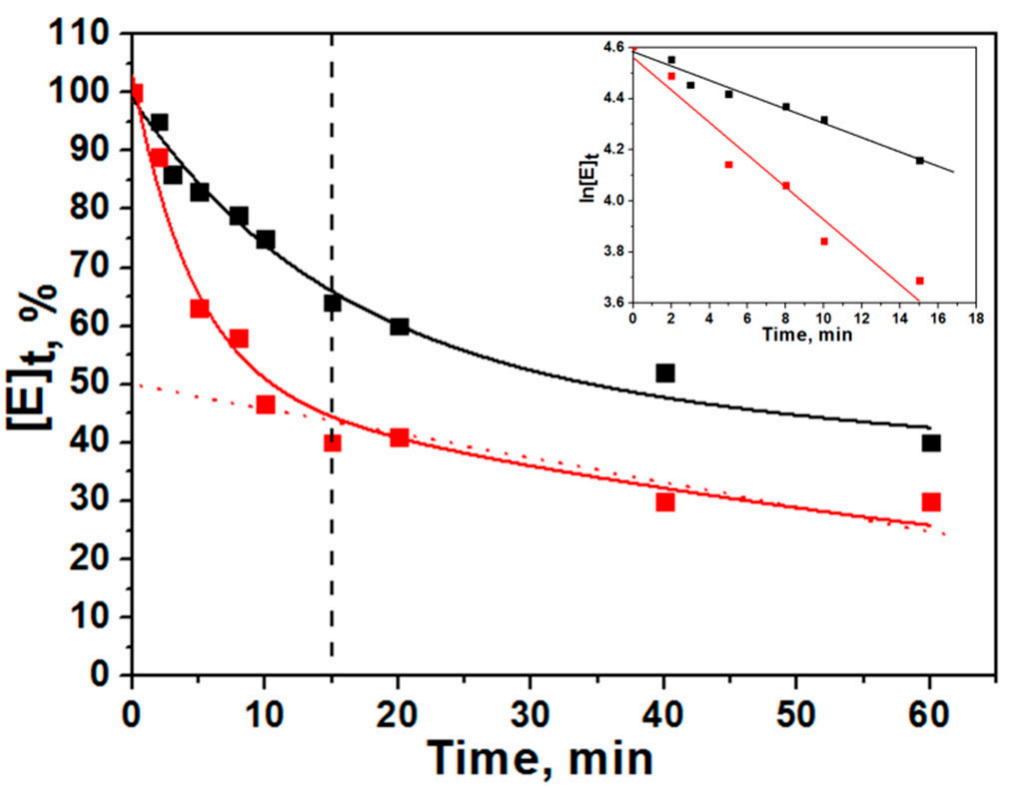
Progressive inhibition of BChE ([E] = 1.7 × 10^−9^ M) as a function of time by echothiophate (5 × 10^−8^ M) as monitored by the decrease in activity with 50 μM BTC (black curve) and 0.2 mM atropine (red curve) under first-order conditions ([E] << K_m_) in 0.1 M phosphate pH 7.0 at 25 °C. [E]_t,0_ = 100% = [E]_0_ + [E’]_0_; [E’]_0_ = 50%. Insert: Ln[E%]) versus time until t = 15 min.

Because echothiophate irreversibly blocks the catalytic serine (S198), a decrease in both activities with time (Figure 4) indicates that BTC and atropine are hydrolyzed by BChEat, the same active site (Ser198). However, the decrease in activity of enzyme showed an important difference, depending on the reporter substrate. While the decrease in activity with BTC was monophasic, as expected, for simple irreversible phosphorylation of the enzyme S198 by OPs such as echothiophate or paraoxon under first-order conditions [OP] >> [E] (cf. Equation (4)), the decrease in activity with atropine as the reporter substrate was biphasic (cf. Equation (5)).

Indeed, in the presence of echothiophate, the decay of residual enzyme activity with time, analyzed by BTC as the reporter substrate, was of the first order, as expected:

(4) $${\left[E\right]}_{t}={\left[E\right]}_{0}e^{-k_{app}t}$$

where [*E*]*_t_* is the percentage of remaining activity at time (*t*) and *k_app_*, the first-order rate constant of phosphorylation at the selected concentration of echothiophate. Semi-log plot data corresponding to Equation (4) gave a linear plot of slope −*k_app_* = −0.03 min^−1^ (insert of Figure 4). On the other hand, with atropine as the reporter substrate, the semi-log plot was biphasic and can only be described by the sum of two first-order processes corresponding to the following:

(5) $${\left[E\right]}_{t}={\left[E\right]}_{0}e^{-k_{app,1}t}+({\left[E\right]}_{0}-Y)e^{-k_{app,2}t}$$

Such a biphasic phosphorylation kinetic process is not unusual and was already described for wild-type and mutant ChEs with different phosphorylating agents [20,21,22]. The first exponential process provides *k_app,_*_1_ = 0.07 min^−1^.

With both reporter substrates, the semi-log plots show the same slope at time t > 20 min (Figure 4), indicating that enzyme phosphorylation follows the same kinetic process beyond this time (with apparent rate constant, *k_app_*), regardless of the reporter substrate used.

It is important to point out the difference in behavior, depending on the reporter substrate. This behavior is related to the existence of two catalytically active enzyme conformational states in slow equilibrium, *E* and *E*’. Such slow conformational equilibria determine the preferential selection of ligands (substrates and inhibitors) by one form [23,24,25]. This leads to hysteretic catalytic behavior of enzymes [26] and slow-binding inhibition kinetics [27]. ChEs were found to display such behaviors with certain substrates, reversible and irreversible inhibitors [28,29]. The decomposition of the phosphorylation biphasic process in two first-order processes (Equation (5) and insert of Figure 4) indicates that each enzyme form (*E* and *E*’) corresponds to 50% of total enzyme and that with 5 × 10^−8^ M echothiophate. Thus, in Equation (5), Y: *E*’ = 50% of total enzyme. The linearity of semi-log plot with BTC (Figure 4) as the reporter substrate is due to the fact that both enzyme forms are equally hydrolyzing BTC regardless of the reactivity of each form with echothiophate, so that the remaining activity of the active enzyme, *E_t_* + *E_t_*’, is constant over the whole timescale. On the other hand, although BChE showed no hysteretic behavior with atropine as the substrate, i.e., no lag or burst preceding the steady-state phase (cf Figure 2), the biphasic behavior with atropine as the reporter substrate revealed that form E is more susceptible to phosphorylation by echothiophate than form *E*’ (in Equation (5), *k_app,_*_1_ > *k_app,_*_2_). The interpretation of this behavior in terms of reporter substrate dependence (preferential selection) is the simplest. We cannot rule out a more complex explanation. In particular, it has recurrently been described that OP molecules may bind reversibly to the peripheral anionic site of ChEs and that the occupation of this site modulates the enzyme reactivity through an induced conformation change [20,30,31,32]. Such an alternative explanation is unlikely because it implies a long-lived OP-induced conformational change persisting after the dissociation of the OP caused by the dilution step at each time-point.

Thus, without further investigation, the substrate dependence hypothesis remains the most straightforward. Anyway, with both substrates, BTC and atropine, it is important to point out that, after 15 min of reaction with echothiophate, the progressive inhibition plots became parallel (Figure 3). This indicates that the enzyme form *E* was fully phosphorylated in about 10–15 min, and that the phosphorylation process continues for the form *E*’ and that its activity decays at the same rate (*k_app_*) as that for the activity reported with BTC. This clearly indicates that hydrolysis of both substrates takes place at the same site (S198). If the active sites were distinct, the remaining activity with both substrates would have generated divergent inactivation progress curves. 

### 2.3. Molecular Docking Simulations

**Figure 5:**
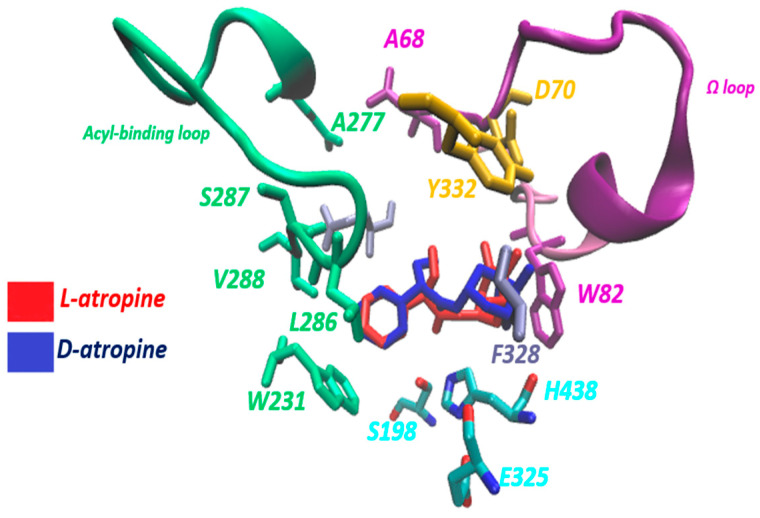
Side view of the active site gorge in human BChE in which D and L atropine are complexed and represented in red and blue color. Crucial residues in the Peripheral Anionic Site (PAS) and Catalytic Anionic Site (CAS) are shown. The Ω-loop is shown in purple, and the acyl-binding pocket (ABP) loop is represented in dim green. H-bonding is observed between PAS residues Y332 and D70 (depicted in dim orange), which play a role in controlling the entrance of the active site gorge. The catalytic triad, consisting of residues S198, H438, and E325, is displayed with atoms C in cyan, N in blue, and O in red. Hydrogen bonds connect the catalytic triad residues (S198…H438…E325) and PAS residues (D70…Y332). The above image was prepared by using visual molecular dynamics software VMD 1.9a51 [33].

In the conducted molecular docking simulations, a comprehensive analysis of the binding interactions between D and L atropine and the active site of human BChE revealed noteworthy insights. Strikingly, the conformations of both D and L atropine were found to be remarkably close in the BChE binding site, suggesting a similar mode of interaction (Figure 5).

**Figure 6:**
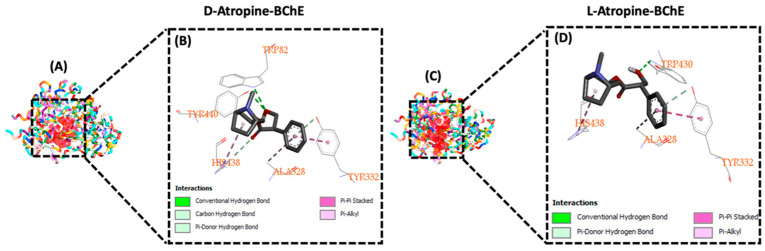
D-atropine interacts with the binding site of the BChE (**A**, **B** enlarged part of **A**). L-atropine interacts with amino acids in the binding site of BChE (**C**, **D** enlarged part of **C**). The color of interactions represents the type of bond formed. The interaction figures were generated by using BIOVIA Discovery Studio Visualizer Software, version 21.1.0.20298, Dassault Systems BIOVA, https://www.3dsbiovia.com/ accessed on 22 January 2024.

Quantifying the binding affinities further underscored the strong association between atropine isomers and BChE. Specifically, D-atropine exhibited a binding affinity of −7.90 kcal/mol, and L-atropine demonstrated a higher binding affinity of −8.68 kcal/mol. These findings collectively point to the high affinity of both isomers to BChE as potent reversible inhibitors of BChE. A detailed examination of molecular interactions sheds light on the specific binding modes adopted by D and L atropine within the BChE binding site. For D-atropine, interactions with the Ω-loop and acyl binding loop were observed, facilitated by amideπstacked, π-alkyl, and π-πT-shaped interactions. The resulting complex, depicted in Figure 6A,B, visually represents the intricate molecular interactions formed by D-atropine with the BChE binding site. Similarly, L-atropine showed interactions with the Ω-loop and the acyl binding loop, relying on alkyl, carbon–hydrogen bond, π-alkyl, and π-πT-shaped interactions. The visual representation of the BChE complexed with L-atropine (Figure 6C,D) vividly illustrates the molecular intricacies of these interactions. To enhance the clarity, certain details, such as the amino acids involved in van der Waals interactions with adjacent amino acids, were omitted from the figures. The color scheme employed in the figure legend delineates the various types of bonds formed during molecular dynamics simulations, providing a comprehensive understanding of the interactions contributing to the strong affinity between the atropine isomers and BChE. These findings offer valuable insights into the potential pharmacological relevance of both D and L atropine as effective ligands of BChE.

### 2.4. Molecular Dynamics Simulations and Free Energy Perturbations

**Figure 7:**
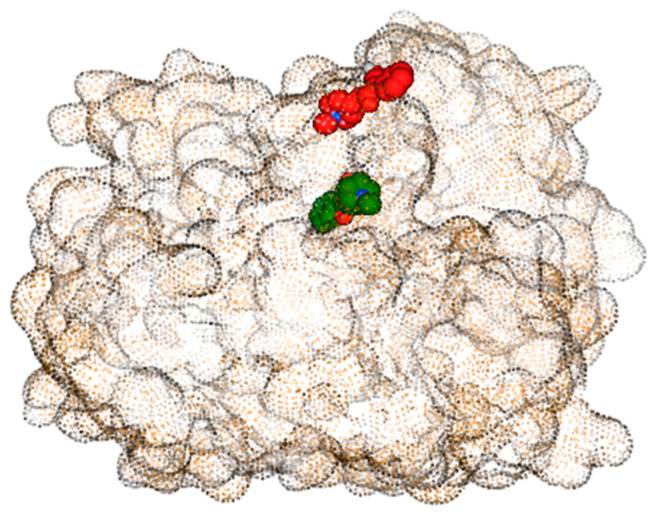
Initial and final lambda states of D-atropine. The lambda_01 in green represents the coupled state of D-atropine, and the lambda_0 state of D-atropine, represented in red, is the decoupled state of D-atropine.

Free energy perturbation (FEP) is a sophisticated computational method rooted in the principles of statistical mechanics, employed within the realm of computational chemistry to calculate nuanced free-energy differences through the integration of molecular dynamics simulations. In essence, FEP serves as a powerful tool that leverages statistical mechanics to explore and quantify the energetic disparities between distinct molecular states. By integrating the principles of thermodynamics into the dynamic framework of molecular dynamics simulations, FEP allows us to discern the subtle variations in free energy that characterize different conformations or chemical environments. This method is particularly valuable in elucidating the thermodynamic consequences of perturbations, such as changes in molecular structure, from the initial lambda and final lambda states represented in Figure 7. Through a meticulous exploration of the potential energy landscape, FEP provides a detailed understanding of how alterations in a system manifest as variations in free energy, shedding light on the energetically favorable or unfavorable transformations that molecules may undergo. 

**Figure 8:**
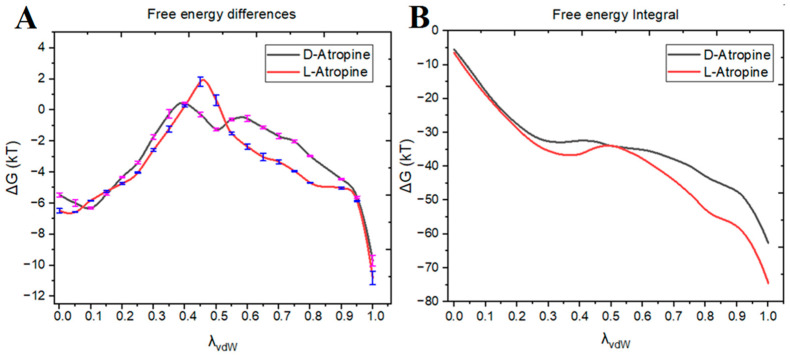
Relative free energy differences for each interval of λ (i.e., between neighboring Hamiltonians) for isomers of atropine represented in the legend box (**A**); and with the cumulative ΔG as a function of λ, the point at λ = 1 corresponds to the sum of ΔG from λ vector 0 to λ vector 1 (**B**).

Utilizing the Bennett Acceptance Ratio (BAR) method in our computational study, we successfully determined the free energy differences associated with the interaction of D- and L-atropine with BChE. The BAR method yielded a computed free energy of binding of −37.28 ± 0.24 kcal/mol for D-atropine and −44.21 ± 0.16 kcal/mol for L-atropine. The free energy differences in each simulation for D&L-atropine are shown in Figure 8. The discerned free energy disparities draw attention to a notable distinction between the two enantiomers. Specifically, L-atropine exhibited a slightly lower free energy compared to D-atropine, signifying a subtle yet significant difference in binding energetically with BChE. This observed contrast in free energy values points towards an intriguing phenomenon: BChE displays enantioselectivity towards D-atropine and L-atropine. Enantioselectivity refers to the preferential interaction of the enzyme with one enantiomer over the other. The more negative free energy associated with L-atropine suggests a stronger binding affinity compared to D-atropine. The implication of a more negative free energy for L-atropine underscores its potential as a better ligand for BChE, indicating a more favorable and energetically favorable interaction. This finding has significant implications, not only in understanding the molecular interactions governing the binding process and the hydrolysis reaction of atropine but also in the potential development of pharmacologically relevant compounds with an enhanced efficacy based on observed enantioselectivity. In summary, the application of the BAR method not only quantified the free energy differences between D- and L-atropine but also revealed critical insights into the enantioselectivity of BChE. The more favorable binding of L-atropine underscores its potential as a promising candidate for further exploration in the development of novel BChE inhibitors and substrates of medical interest [6]. Moreover, although this work cannot provide an answer about the reactivity of both atropine isomers with the active site serine of human BChE, the better affinity of the L-isomer suggests that this isomer is capable of forming the productive complex more easily than the D-isomer. BChE-catalyzed hydrolysis of atropine using pure stereoisomers should provide a definitive answer about the enantiomeric preferential selectivity of the enzyme for atropine.

## 3. Discussion

Atropine was found to be a substrate of BChE, and sensitivity to the OP echothiophate revealed that hydrolysis takes place on S198 of the enzyme CAS. The hydrolysis kinetics was recorded only under first-order conditions, i.e., at an atropine concentration much lower than *K_m_*. This provided a value of *k_cat_*/*K_m_* = 7.7 × 10^4^ M^−1^ min^−1^. Under the same conditions of pH, buffer, and temperature, the value of *k_cat_*/*K_m_* for BTC, the best substrate of BChE, is 1.3 × 10^9^ M^−1^ min^−1^ [19]. Thus, atropine is a poor substrate of BChE, with a specificity constant about 5 orders of magnitude less than for BTC. Rough estimates of *K_m_* and *k_cat_*, ranging between 0.3 and 3.35 mM and between 11.5 and 38.5 min^−1^, respectively, fit with experimental observations, indicating a modest affinity and a slow hydrolysis rate that is limited by the acylation step.

Molecular docking simulations serve as powerful tools in understanding the binding interactions between ligands and target proteins. Simulations involving D and L atropine with human BChE indicated a comparable mode of interaction. The binding affinities for D-atropine (−31.68 kcal/mol) and L-atropine (−34.84 kcal/mol) suggest that both isomers have the same potential to bind BChE for making complexes. The comprehensive figures provided valuable insights into the molecular intricacies of interactions. Moving beyond docking simulations, the study delved into molecular dynamics simulations and free energy perturbations (FEPs). By integrating FEP with molecular dynamics simulations, the study aimed to discern the subtle variations in free energy, providing a detailed understanding of how alterations in the system manifest as variations in free energy. Then, FEP stands as a cornerstone in computational chemistry, facilitating the accurate prediction and understanding of free-energy differences. This approach ultimately contributes to paramount advancements in drug discovery. 

The Bennett Acceptance Ratio (BAR) method was employed to determine the free energy differences associated with D-atropine and L-atropine interactions with BChE. The results indicated a slightly lower free energy for L-atropine compared to D-atropine, implying the enantioselectivity exhibited by BChE. The observed more negative free energy for L-atropine underscores its potential as a better ligand for BChE, suggesting a more favorable and energetically favorable interaction. Both computational strategies, namely molecular docking simulations and free energy perturbations, demonstrated that atropine isomers fit within the binding site of BChE. However, a more pronounced increase in binding affinity, as indicated by the negative values derived from the free energy perturbation analysis, confirmed that these isomers are potential inhibitors of BChE. This statement fits with old experimental inhibition kinetic data (see [13] and cited works in [6]). These findings were further contextualized by comparison with the related tropane ester of benzoic acid, cocaine, that reacts with the active site serine, S198, and undergoes hydrolysis [34,35]. Notably, the computational analysis did not identify any interactions between the atropine isomers and S198, which are responsible for BChE-catalyzed hydrolysis of esters substrates. This lack of interaction suggests a different mechanism of reversible inhibition. However, our computational insights are in strong concordance with the experimental kinetic data, revealing that atropine undergoes a very slow hydrolysis upon the action of BChE. This suggests that, unlike cocaine, atropine isomers interact with BChE in a manner that does not prominently involve the formation of productive complexes with S198, reinforcing their potential as BChE reversible inhibitors.

Although both the affinity (based on estimated *K_i_* [13] and estimated *K_m_*), turnover number and bimolecular rate constant of BChE for atropine are slow and are owed to the average concentration of the enzyme in plasma (i.e., 5 mg/L or 1.47 × 10^−8^ M), with [*A*] << *K_m_* and [*E*] << [*A*], the degradation of atropine (A) in the bloodstream by BChE occurs under first-order conditions (Equation (1)) in which (*k_cat_*/*K_m_*). [*E*] is the first-order rate constant of degradation (*k_deg_*). Thus, the contribution of plasma BChE to the decay of atropine concentration, [*A*]_t_, in the bloodstream can be described by Equation (6):

[A]_t_ = [A]_0_ exp(−*k_deg_*·*t*) (6)

The constant *k_deg_* is very low, = 0.001 min^−1^. Then, the BChE-catalyzed degradation of atropine in blood does not significantly contribute to the rapid decay of atropine concentration in the bloodstream. Thus, the fast pharmacokinetics of the drug results from the action of hepatic enzymes, in particular, carboxylesterases and cytochromes P450, glucuronide formation, and large urinary excretion of non-metabolized drug [2,3,36].

Therefore, it is unlikely that BChE plays a role in the hydrolysis of atropine under normal therapeutic conditions, where the concentration of atropine in plasma is much less than *K_m_*. BChE contribution might significantly affect the plasma metabolism of atropine under sustained long-term atropinization. However, high doses of atropine administered for long periods of time are needed mostly for post-exposure treatment of OP or carbamate poisoning, where plasma BChE is irreversibly inhibited by the toxicant. Thus, under these specific conditions, neither plasma and cellular BChE nor liver carboxylesterases play a role in catabolism of atropine.

In the extreme case of chemical war, where OPs were used by Iraq against Iranian troops, up to 200 mg (0.28 mmoles) of atropine in a 10–15 min period *pro re nata* was intravenously administered by Iranian medics to chemical casualties [37]. Assuming a constant atropine concentration in such a short period of time, the maximum plasma concentration of atropine in a 70 kg human (2.8 L plasma) would have thus been 0.1 mM after intravenous injection. This concentration was still lower than K_m_ of BChE for atropine, and in the case of acute OP poisoning, plasma BChE would have been fully irreversible inhibited and, thus, unable to degrade atropine. Therefore, *reductio ad absurdum*, this led us to the conclusion that, under the current conditions of medical use of atropine (maximum dose of 0.3–3 mg i.v), providing at injection time (t_0_) at maximum atropine concentration in plasma of 4.3 × 10^−5^ M, about one or two orders of magnitude below the estimated *K_m_* of BChE for atropine, plasma BChE has a non-significant role in the metabolism of atropine in human.

## 4. Materials and Methods

### 4.1. Chemicals and Enzyme

Butyrylthiocholine iodide (BTC) and dithio-bis-nitrobenzoic acid (DTNB) were purchased from Sigma-Aldrich (Saint Louis, MO, USA). Stock solutions of BTC (0.1 M) was in water and stored at −20 °C. Echothiophate iodide was from Biobasal AG (Basel, Switzerland). Stock solution of 0.1 M echothiophate was in solution in water and stored at −20 °C. Atropine sulfate racemic was from OAO Dalkhimpharm (Khabarovsk, Russian Federation). Stock solution (1 mg/mL, 1.43 mM) was in water.

Human BChE tetrameric form (MW = 340 kDa) that was highly purified from human plasma Cohn fraction IV-4 [38] was a gift from Dr. O. Lockridge (UNMC, Omaha, NE, USA). The enzyme was diluted in 0.1 M sodium phosphate buffer pH 7.0 to an activity of 45 units/mL, with 1 mM BTC as the substrate, at 25 °C (one unit corresponds to the number of micromoles of BTC hydrolyzed per minute).

### 4.2. Enzyme Titration

The diluted enzyme was titrated according to the method of Leuzinger [39], using echothiophate iodide as the titrant. The active site concentration of this preparation was 3.4 × 10^−8^ M. During the titration processes, enzyme activity was checked using the method of Ellman [40], with 1 mM BTC in 0.1 M phosphate buffer pH 7.0 at 25 °C, in the presence of DTNB (0.01 M), which functioned as the thiocholine-reacting chromogenic dye. 

### 4.3. Steady-State Hydrolysis of Racemic Atropine Sulfate

BChE-catalyzed hydrolysis of atropine was studied in 0.1 M phosphate buffer, pH 7.0 at 25 °C. Atropine concentration ranged from 0.025 to 0.20 mM. The highest concentration of atropine we used, 0.2 mM, was selected because it corresponds approximately to the double of the highest concentration of atropine in human plasma after injection of the highest possible doses of atropine in human medicine. The enzyme concentration in most assays was 0.17 × 10^−8^ M. However, for the study of the dependence of hydrolysis rate on enzyme concentrations, enzyme concentration was increased up to 5.1 × 10^−8^ M. Hydrolysis was spectrophotometrically monitored in UV at 240 nm by following the decrease in absorbance of atropine (release of product *P*_2_, 3-hydroxy-2-phenylpropanoic acid), as for the classical assay of BChE-catalyzed hydrolysis of benzoylcholine, where the release of product P_2_, benzoic acid is also monitored at 240 nm [41]. The difference in the absorptivity constant at 240 nm (∆ε) between atropine and product *P*_2_ was 418 M^−1^ cm^−1^. The double-beam spectrophotometer was a Shimatzu UV-visible 800, Peltier-thermostated apparatus.

Catalytic parameters were determined from linear or non-linear curve fitting of kinetic data, using the software OriginPro 8.5 (Originlab Co., Northampton, MA, USA).

### 4.4. Irreversible Inhibition of BChE-Catalyzed Hydrolysis of Atropine and Butyrylthiocholine by Echothiophate

Because the catalytic residue of BChE, S198, is the target of irreversible inhibitors like organophosphates (OPs), the site responsible for the hydrolysis of atropine was probed by an OP.

Time-dependent irreversible inhibition of BChE by the organophosphoryl ester echothiophate was performed under first-order conditions, according to the sampling method of Aldridge [42], in 0.1 M phosphate buffer pH 7.0 and 25 °C. The incubation of the enzyme with the OP for fixed times was followed immediately by the extensive dilution of samples in a cuvette for measuring the remaining activity. First, to determine the echothiophate concentration to be used, in a preliminary experiment, different concentrations of echothiophate, ranging from 10^−7^ to 10^−8^ M, were tested. The enzyme concentration was 1.7 × 10^−9^ M so that inhibition was always performed under first-order conditions ([*E*] << [echothiophate]). For this preliminary experiment, the reference ester substrate was BTC, and the residual activity of BChE versus time was monitored by the method of Ellman [40]. The selected concentration in echothiophate was 5 × 10^−8^ M; it provided 50% of irreversible enzyme inhibition in less than 10 min with 1 mM BTC, the standard concentration close to V_max_. Then, BChE was inhibited by this echothiophate concentration, and the progressive decrease in activity of the enzyme versus time was monitored with both substrates, using 0.050 mM BTC and 0.2 mM atropine, respectively. At time 0, the enzyme activity with both substrates was 100% = [*E*]_0_. The first-order decay of enzyme activity with both substrates was analyzed using the software Origin.

### 4.5. Molecular Modeling

#### 4.5.1. BChE Modeling and Molecular Docking Simulation

The 3D structure of Human BChE tetrameric form (MW = 340 kDa) was sourced from https://www.rcsb.org/ (accessed on 22 January 2024). The quaternary structure of the enzyme is composed of four identical chains [14]. The missing amino acids in the X-ray crystallographic structure were filled by using Swiss-model https://swissmodel.expasy.org/ (accessed on 23 January 2024). [43]. Both structures were sketched by using Avogadro software Version: 21.1 [44] and were minimized by the steepest-descent method. Initially, the protein underwent preparation steps under the Discovery Studio software (DS, 2021). The water molecules were removed and saved in the pdb format. 

Molecular docking of atropine was conducted using the PyRx software version 0.8 [45]. The PrankWeb online server available at http://prankweb.cz/ (accessed on 23 January 2024) [46] was used to identify the binding pocket of BChE. The BChE was imported to the PyRx software and converted to a readable format. The atropine was imported into PyRx through the insert module. The atropine isomers were converted to the pdbqt format following minimization by using the mmff94 force field through the steepest method. Next, the grid was built around the binding site with dimensions x, y, and z of 132.68, 116.08, and 36.835, respectively, with grid centers x, y, and z being 21.88, 26.47, and 22.45 Å. The exhaustiveness score was set to 8. The Lamarckian genetic algorithm (LGA) used was adopted to generate the binding poses with random seed search [47,48]. The interactions established with atropine were identified by using BIOVIA Discovery Studio Visualizer Software, Version 21.1.0.20298, Dassault Systèmes, 2021 [49]; and the figures were prepared by VMD software VMD 1.9.4a51 [33].

#### 4.5.2. Molecular Dynamics Simulations 

In GROMACS 22.04., a popular molecular dynamics simulation package [50,51], solvation methods were used to model the interaction of atropine isomers with BChE. The choice of solvation method is crucial for obtaining realistic simulation results. The free energy perturbation (FEP) method was used for free energy solvation calculations. The docked complexes were used as initials for the FEP calculations. The topology for BChE was created by using amber99sb-ildn.ff [52,53], and the topology for atropine isomers was written through a t-leap server and GAFF [54,55]. Both topologies were merged to form a complex. The complex was placed in the dodecahedron box, 1 nm from the SPC boundaries. The system was solvated by applying 20204 SOLs. Further solvated complexes were minimized and the global equilibration was conducted through a series of equilibration steps, i.e., NVT (constant number of particles, volume, and temperature) and NPT (constant number of particles, pressure, and temperature) simulations [56]. The cutoffs for non-bonded interactions, rlist and nstlist, were set to be 1.0 nm and 10 steps. The electrostatic interactions were managed by the PME method for efficient calculations [57]. The all-bonds constraints were instructed to constrain bond lengths of the molecule to maintain initial values. The temperature was kept constant at 300 K by applying a v-rescale thermostat with a coupling constant of 0.2 ps. The pressure was kept fixed at 1 bar using the Berendsen barostat with a coupling constant of 5 ps. The hydrogen bond network was handled by the LNICS algorithm, and the Verlet leapfrog algorithm was employed to integrate the equation of motion.

In our computational approach, we employed the concept of lambda points to systematically decouple the ligand from the coupled system—specifically, the atropine isomers from BChE. A set of 20 lambda points, ranging from 0 to 1, with increments of 0.05, was strategically chosen to comprehensively explore the free energy change during the decoupling process. Each lambda point represents a specific stage of ligand decoupling, with 0 corresponding to the fully decoupled state, and 1 representing the fully coupled state. These 20 lambda points play a pivotal role in characterizing the gradual transition from the decoupled to the coupled state. The population of these lambda points facilitates the calculation of the free energy change associated with the atropine isomers’ interaction with BChE across the entire decoupling process. It is crucial to emphasize that this meticulous decoupling process is integral to establishing a stable starting point for subsequent free energy perturbation simulations. By systematically transitioning the system through these lambda points, we ensure that the simulation trajectories effectively explore the conformational landscape, providing a foundation for accurate and reliable free energy calculations. During the production simulation phase, each lambda point, from the starting state (lambda_0) to the ending state (lambda_20), is rigorously simulated to gather the necessary data for subsequent free energy calculations. This step-by-step exploration allows for the thorough sampling of the ligand’s behavior within the complex, capturing the intricacies of the interaction between atropine isomers and BChE at different stages of coupling. In summary, the inclusion of 20 lambda points in our methodology is a systematic and essential approach to unraveling the free energy changes associated with the decoupling of atropine isomers from BChE. These lambda points serve as critical milestones in the exploration of ligand–protein interactions and contribute to the overall robustness of our free energy perturbation simulations. The Bennett Acceptance Ratio (BAR) method with the appropriate flags was employed to analyze the results of FEP [58].

## 5. Conclusions

In conclusion, it is accepted that plasma butyrylcholinesterase (BChE) is a pharmacologically important enzyme that plays a role in the metabolism of numerous ester drugs [6]. However, the present work demonstrates that the participation of plasma and cellular BChE in the degradation of atropine is negligible even when the highest possible doses of atropine are administered. Same conclusions can be drawn regarding the hydrolysis of the related alkaloid, hyoscine (scopolamine). Lastly, even though that injection of huge doses of human BChE as a bioscavenger can be considered for the detoxification of poisonous ester substrates like succinylcholine, heroin, or cocaine [6], the slow *k_cat_*/*K_m_* of BChE for atropine indicates that it would not help to catalytically scavenge high doses of atropine and other antimuscarinic tropanol esters like hyoscine or the bulkier 3-quinuclidinyl benzylate, a banned incapacitating agent.

## Data Availability

Data are contained within the article.

## Abbreviations

A	atropine
BAR	Bennett Acceptance Ratio
BChE	butyrylcholinesterase
BTC	butyrylthiocholine
CAC	catalytic active center
CES	carboxylesterase
ChE	cholinesterase
FEP	free energy perturbation
MD	molecular dynamics
OP	organophosphate
PAS	peripheral anionic site
